# Cluster randomised controlled trial for service delivery redesign of primary care for people with diabetes: study protocol

**DOI:** 10.1136/bmjopen-2025-111459

**Published:** 2026-03-18

**Authors:** Agustina Mazzoni, Javier Roberti, Marina Guglielmino, Facundo Jorro-Baron, Yanina Mazzaresi, Andrea Falaschi, Jorgelina Alvarez, Luz Gibbons, Hannah H Leslie, Cecilia Silva, Patricia J Garcia, Ezequiel Garcia-Elorrio

**Affiliations:** 1 Health Care Quality and Patient Safety Department, Institute for Clinical Effectiveness and Health Policy, Buenos Aires, Argentina; 2 Qualitative Research Unit, Institute for Clinical Effectiveness and Health Policy, Buenos Aires, Argentina; 3 Department of Health Promotion and Prevention, Ministry of Health, Mendoza, Argentina; 4 Department of Epidemiology, Ministry of Health, Mendoza, Argentina; 5 Health Technology Assessment Agency, Ministry of Health, Mendoza, Argentina; 6 Unit of Statistics, Data Management and Information Systems, Institute for Clinical Effectiveness and Health Policy, Buenos Aires, Argentina; 7 University of California San Francisco, San Francisco, California, USA; 8 Obra Social de Empleados Públicos de Mendoza, Mendoza, Argentina; 9 School of Public Health, Cayetano Heredia Pervuvian University, Lima, Peru

**Keywords:** Diabetes Mellitus, Type 2, Primary Health Care, Implementation Science, Randomized Controlled Trial

## Abstract

**Introduction:**

Strong primary healthcare enhances resource efficiency and resilience. Type 2 diabetes poses a growing global health challenge, with Argentina’s healthcare system struggling to detect and manage the disease effectively. Many patients bypass primary healthcare for secondary facilities, undermining continuity of care and increasing costs. Following a diagnostic process in collaboration with policymakers, we propose evaluating a redesigned primary care model consisting of codesigned evidence-based implementation strategies to improve type 2 diabetes management in Mendoza, Argentina.

**Methods and analysis:**

This is an efficient, parallel-arm cluster randomised controlled Hybrid Type II trial with 12 clusters (administrative areas with 2–3 health facilities) allocated 1:1 to control (usual care) or intervention. In phase I, we will codesign, pilot and refine an implementation strategy package. In phase II, we will conduct the trial: 9-month baseline data collection, 15-month intervention and 6-month sustainability period. We will enrol a cohort of 396 patients with type 2 diabetes at primary healthcare centres and follow them for 12 months during the intervention and 6 months sustainment using routine clinical records and patient surveys. In phase III, we will conduct analysis, report and disseminate findings. The primary outcome will be a composite outcome including glycaemic control (glycated haemoglobin (HbA1c) <8%); blood pressure control (<140/90 mm Hg) and statin prescription (limited to patients ≥40 years) from clinical records. The primary analysis will compare the proportion of patients achieving this composite clinical outcome between the trial arms at the end of the study. Secondary analyses include assessing patient experience and primary healthcare engagement; testing the implementation strategies’ impact on service use patterns, system competence, user confidence and cost per visit; exploring inequalities by sociodemographic factors; and assessing patient empowerment. We will use all available data from all randomised clusters and conduct all analyses on the intention-to-treat population, regardless of intervention adherence.

**Ethics and dissemination:**

All study activities will comply with national and international ethics guidelines, presenting minimal risk to participants. The protocol was submitted and approved by the local independent ethics committee at the Mendoza Ministry of Health (Consejo Provincial de Evaluación ética en investigación en Salud-Provincial Health Research Ethics Review Board, Reference number: 149/2024). Facility-level permission will be obtained for participation and sharing of deidentified data. Written informed consent will be required from study participants, who will receive information on the study’s purpose, procedures, risks and benefits. Dissemination activities and outputs will include writing and submitting manuscripts for publication; writing policy briefs to support strategy implementation in other regions or countries; and tailoring outputs for patients, clinicians and researchers. We anticipate that improvements in disease management and patient experience will have clinical and economic benefits related to reduced usage of secondary-level and tertiary-level facilities, lower cost per visit and a reduced number of clinical events related to diabetes.

**Trial registration number:**

ISRCTN63277390.

**STRENGTHS AND LIMITATIONS OF THIS STUDY**

- This study integrates the Implementation research logic model and the normalisation process theory to support the design and evaluation of strategies. This dual framework approach enhances our capacity to understand whether the strategies improve care and how and why they do so in complex, real-world settings.
- The inclusion of community engagement throughout all phases of the study aims to ensure relevance and enhance the sustainability of the strategies to be implemented.
- As far as we know, this study will be among the first cluster randomised controlled trials in Latin America to evaluate the implementation of a co-designed care model for chronic diseases in the public primary healthcare system.
- Implementing this study in an Low- and middle-income countries (LMIC) is complex and influenced by dynamic factors such as political shifts, workforce constraints, competing policy priorities and resource limitations.
- Findings from a single province in Argentina may have limited generalisability to other settings without careful adaptation to local health system structures and sociocultural dynamics.

## Introduction

 Strong primary healthcare (PHC) saves lives, improves resource use efficiency and makes health systems more resilient and effective for the population.^1^ Functional PHC systems should be able to prevent and detect prevalent and impactful non-communicable diseases (NCDs) such as diabetes and to manage ongoing diseases.^2^ Service delivery redesign (SDR) is an innovative, system-level framework for improving care delivery defined as the reorganisation and strengthening of existing services and care pathways to maximise quality care and optimise health outcomes.^3^

Latin American countries have been at the forefront of achieving universal health coverage for their populations and working to provide equitable person-centred care.^4^,^6^ However, health system quality in this region is inadequate to deliver on the promise of universal health coverage, resulting in incomplete detection, insufficient control of chronic conditions and high out-of-pocket costs.^7^ There are also a few systematic efforts to give patients a voice about their opinions and experience with the care they receive.^8^ Argentina, an upper-middle-income country in Latin America, has a primarily publicly financed health system: 60% of the population is covered by the social security sector run by labour unions and at least 36% by government healthcare, with the remaining 4% covered by private insurance.^9^ Dependence on public healthcare will only continue to increase as economic turmoil fuels rising unemployment.

Type 2 diabetes mellitus (T2D) is an increasing global health burden. In 2019, diabetes was the direct cause of 1.5 million deaths worldwide.^10^ In addition to the cost of treatment for T2D and its complications, which is a significant burden for national health services, T2D and other NCDs heavily impact productivity, particularly concerning lost or reduced working days due to illness.^11^ Central and South American countries spend an average of 18.4% of their total health expenditure on diabetes.^12 13^ Diabetes detection and control are primarily the responsibility of the non-private schemes.^14 15^ To date, Argentina’s PHC system has failed to meet this challenge; T2D detection and control coverages are low, and those in treatment often bypass PHC for secondary facilities.^16^ Of the estimated 12.7% of the national population living with T2D, only 50% are aware of living with their diagnosis, and only 40% of these individuals have achieved metabolic control (HbA1c <7%), leaving 80% of cases uncontrolled.^17 18^

The Quality Evidence System Transformation Network (QuEST Network) provides policymakers with evidence and tools for health system improvement. QuEST-Latin America and the Caribbean leads regional research and dissemination.^19^ Collaboration with the government of the Argentinian province of Mendoza led to efforts to address post-COVID-19 health challenges. Together, we conducted a comprehensive assessment of PHC system quality, focusing on NCDs and patient experience. This diagnostic assessment included a population-based survey of 1220 respondents, a cohort study of patients with diabetes, analysis of routine health data to identify care patterns and disease control, and qualitative studies of how NCD patients use PHC services and how providers interact with these patients.^16 20^ Our analysis from this diagnostic process showed significant deficits in patient experience, system competence and trust in the public health system. The study found that accessing care was difficult due to appointment difficulties and long waiting times, and obtaining medication and laboratory tests at secondary centres was also problematic. Financial constraints were significant, with co-payments in the social security sector, transportation costs and lost work revenue. Strategies employed included networking, emergency service use, careful planning and taking loans.^16^ We also conducted a Delphi consensus process with local stakeholders to identify specific recommendations, based on the findings of the diagnostic studies, to improve healthcare services.^21^ Based on this diagnostic process and our established relationship with policymakers and stakeholders in the province of Mendoza, we propose to codesign a new model of care to be tested and evaluated in a pragmatic cluster randomised controlled trial to improve health for individuals with diabetes in Argentina.

In this protocol, we conceptualise the redesigned PHC model itself—built around the national clinical guideline for T2D management—as the core intervention. Our trial tests a package of implementation strategies designed to embed this guideline-based model into everyday practice.

## Methods and analysis

### Aims

The primary objective is to improve disease control in individuals with T2D. Secondary objectives are (a) to improve the care experience, (b) to assess the feasibility and acceptability of the redesigned model and its implementation package, (c) to test the model’s impact on service use patterns, system competence, user confidence and cost per visit and (d) to analyse the impact of the model on potential inequalities by sociodemographic factors.

### Design

**Figure 1:**
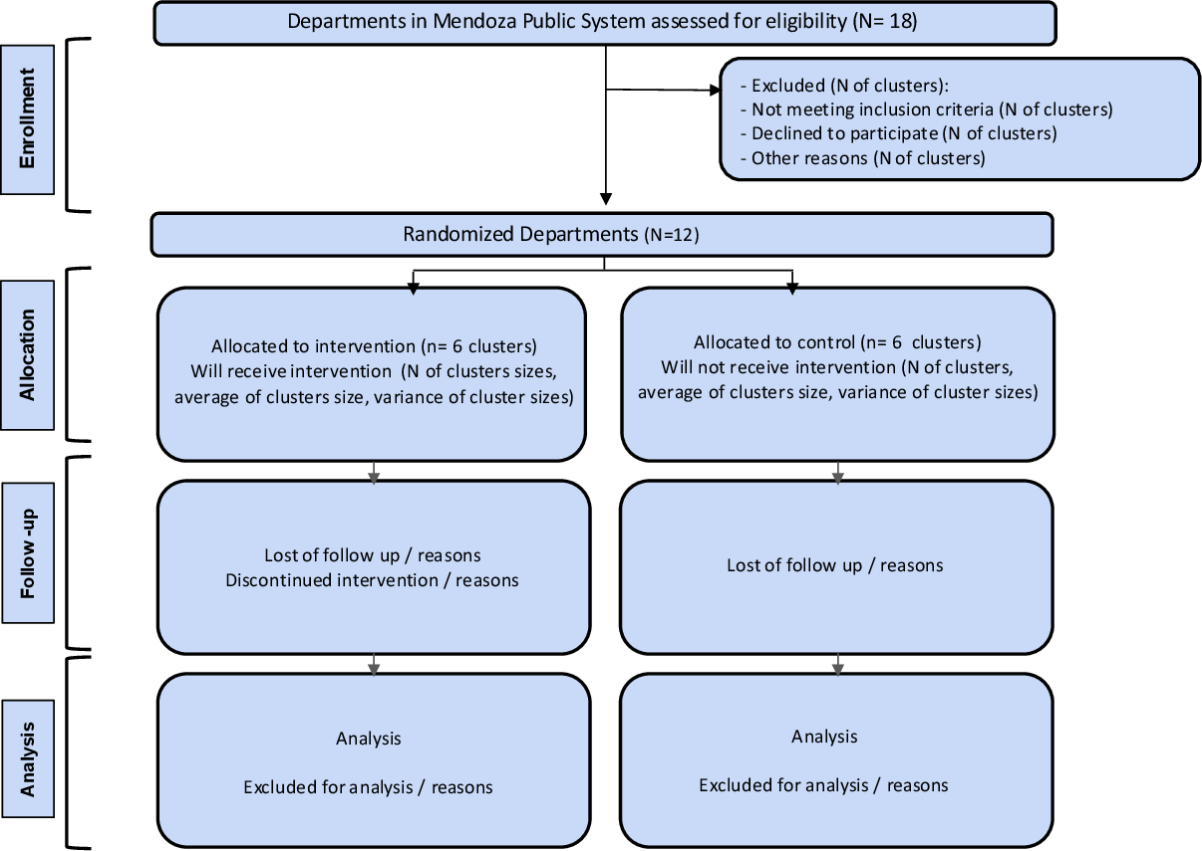
CONSORT flow diagram. CONSORT, Consolidated Standards of Reporting Trials.

**Figure 2:**
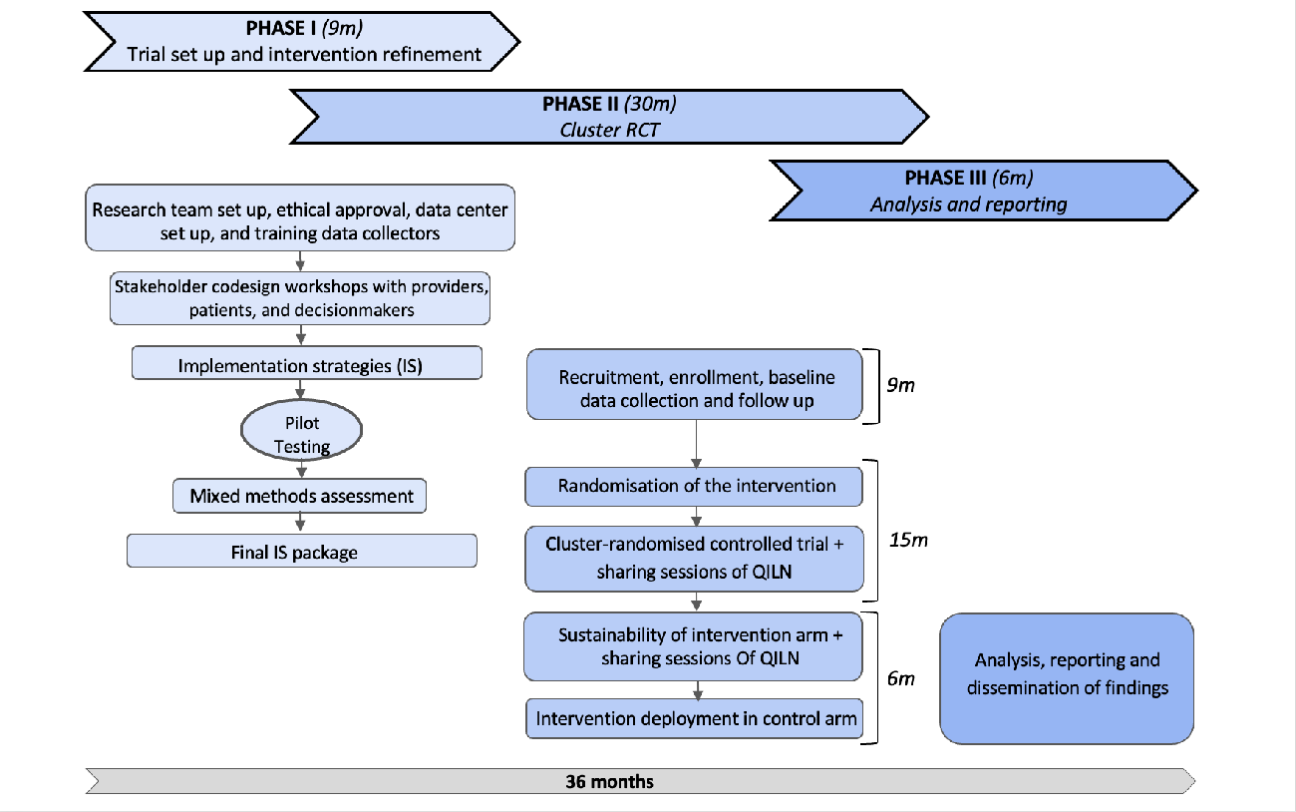
Study overview. QILN, Quality Improvement Learning Network; RCT, randomised controlled trial.

This study is a pragmatic, parallel-arm, cluster-randomised controlled Hybrid Type II trial. 12 provincial health and administrative departments, each comprising 2–3 PHC centres—clusters—will be randomised in a 1:1 ratio to either the intervention or control (usual care) arm (figure 1). The trial will be conducted over 36 months and organised into three overlapping phases (figure 2). Phase I (9 months): Codesign, pilot testing the PHC care model’s refinement and associated implementation strategies. This phase will involve a pilot in two PHC centres, identification of barriers and iterative adaptation of the strategies. Phase II (30 months): Implementation of the trial. This phase includes a 9-month baseline data collection period, a 15-month intervention implementation period, and a 6-month follow-up in the intervention group to assess the sustainability of the implementation strategies. Phase III (6 months): Analysis, reporting and dissemination of findings. The study started on 1 November 2024 and ends on 31 October 2027.

### Patient and public involvement

Community engagement (CE) will be integral to the study. CE is defined as a process of inclusive participation that promotes genuine partnerships among individuals and groups who share geography, interests or experiences, with the aim of addressing issues affecting community well-being.^22^,^24^ This approach is not only ethically imperative but also instrumental in building trust, enhancing participation and promoting the acceptability and uptake of research findings. Four stakeholder workshops will be held annually throughout the study to support CE. These will involve patients, community representatives, healthcare providers and decision-makers. The workshops will serve as a platform to review study progress, promote inclusive dialogue and ensure that the research remains aligned with community needs and priorities. A CE coordinator will facilitate these sessions, with particular attention to the perspectives of underserved populations who experience barriers to healthcare. Workshops will take place in PHC centres and incorporate structured facilitation techniques to encourage diverse participation and equitable voice. Additionally, a stakeholder advisory committee—comprising a community liaison, a social worker and representatives from participating PHC centres—will oversee and guide CE activities. This committee will provide feedback on the implementation process, monitor the responsiveness of the intervention to community input, and contribute to the long-term sustainability of the model of care.

#### Phase I: codesigning and piloting the care model and its implementation strategies

**Figure 3:**
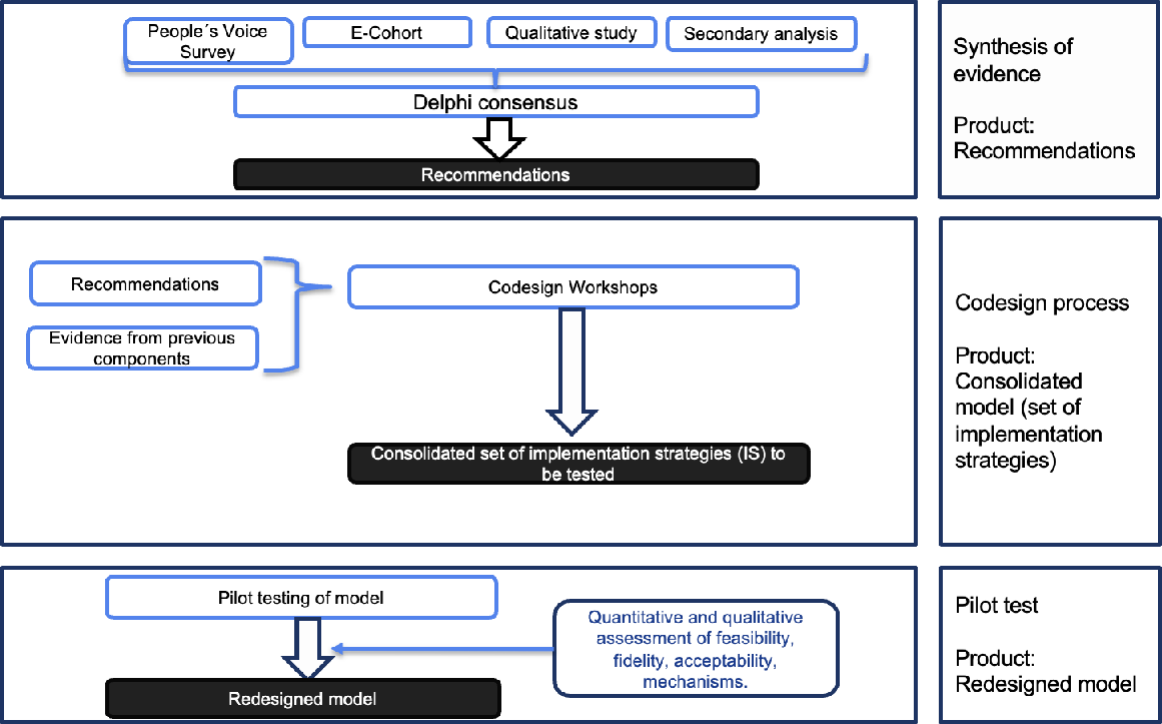
Phase I: model design, refinement and pilot testing.

The purpose of this phase is to codesign a PHC care model and its associated implementation strategies to be tested in a cluster randomised controlled trial, aiming to improve disease management and care experience in patients with T2D. Figure 3 shows the flow chart of this phase.

We define the intervention as the guideline-driven PHC care model for diabetes, and implementation strategies as the theory-informed activities that will support integration of that model into routine practice.

##### Theoretical framework

The study will be guided by three complementary frameworks: the implementation research logic model (IRLM), normalisation process theory (NPT) and principles of CE.

The IRLM will be used to structure the design and evaluation of the implementation strategies. This framework maps the relationships between context-specific determinants, selected implementation strategies, mechanisms of action and outcomes.^25^ It will support a systematic approach to identifying barriers and facilitators, selecting appropriate strategies and articulating how these are expected to produce change. The IRLM is grounded in three core principles: (a) implementation strategies must be tailored to the specific context, (b) strategies operate through distinct mechanisms and (c) implementation outcomes, shaped by these mechanisms, ultimately influence clinical outcomes.^22^

The NPT will complement the IRLM by offering an explanatory lens to understand the processes through which the codesigned care model and its associated implementation strategies become embedded in routine practice.^26^ NPT will be used during the codesign phase to identify factors that may affect stakeholder engagement and operationalisation of implementation strategies.^27^ It will also guide the evaluation of implementation processes. NPT posits that complex interventions are normalised through collective action and shared sense-making. The theory highlights four key constructs: coherence (how stakeholders make sense of the intervention), cognitive participation (engagement and commitment), collective action (how the work is enacted) and reflexive monitoring (ongoing appraisal and adaptation).^26 28^ These constructs will be used to examine how individuals and teams integrate the intervention into existing workflows and cultures. Together, IRLM and NPT will ensure that the development, deployment and evaluation of the implementation strategies are grounded in both theory and context.

##### Procedures

The codesign process will be structured through a series of workshops aimed at developing a new care model for improving diabetes care in PHC settings. The workshops will be held in a venue provided by the Ministry of Health, ensuring a conducive and collaborative environment. A multidisciplinary team, including researchers, a psychologist, a social worker and a drama teacher, will facilitate the sessions. Participants will include patients, healthcare professionals and policymakers, who will be grouped to encourage diverse perspectives in discussions. The workshops will involve interactive activities such as role-playing and user journey mapping to identify barriers and facilitators in diabetes care. The final sessions will focus on prioritising solutions using the IRLM, and then participants will finalise the implementation strategies, ensuring feasibility and alignment with available resources.

##### Piloting the PHC care model and its associated implementation strategies

The PHC care model package will be tested and piloted in two facilities selected by the research team and study advisors, meeting the eligibility criteria for the effectiveness trial but will not be included in the trial. The pilot will last for 4 months. The PHC centre director will organise a team that includes physicians, nurses and a representative from a patient group. We will implement co-designed strategies and monitor the implementation. Throughout the pilot, we will conduct on-site evaluations and surveys with patients and providers to assess the acceptability of the model. After the pilot, we will conduct an implementation evaluation through two focus groups with patients from the participating sites and eight in-depth interviews with healthcare providers to investigate patients’ and providers’ experiences with the implementation, fidelity, acceptability and feasibility. Information obtained through the triangulation of data collected through on-site evaluation, clinical records, surveys, focus groups and interviews will address the implementation outcomes of feasibility, fidelity and acceptability.^29^ Qualitative data from focus groups and interviews will be used to describe the experience and identify the challenges in implementing the model and its strategies. In preparation for the implementation trial, we will refine the model structure, manualised procedures, needed resources and training materials, and finalise data collection tools. As part of the first phase of this study, we will conduct an implementation mapping exercise, systematically linking each strategy to the determinants identified during our diagnostic studies using the Expert Recommendations for Implementing Change (ERIC) taxonomy. These mappings will be captured in our IRLM and refined iteratively, ensuring that every activity directly addresses a known barrier to normalisation.

### Phase II: trial deployment

#### Setting

The trial will be implemented in PHC centres across the province of Mendoza, Argentina, which serve approximately 45% of the population, primarily individuals from socioeconomically disadvantaged backgrounds. The province is administratively divided into 18 departments. The unit of randomisation (cluster) will be the department. 12 departments will be selected, each contributing at least two PHC centres. Clusters will include two or three PHC centres providing care to ≥50 individuals diagnosed with T2D per cluster. Centres involved in the pilot phase will be excluded from phase II.

#### Study participants

The study population will include adult patients with T2D who receive care through the public healthcare system. Eligibility criteria are age ≥18 years, a confirmed diagnosis of T2D for at least 1 year, residency in Mendoza province and receipt of care at one of the participating PHC centres. We will construct a longitudinal cohort of 396 patients with T2D in both arms, enrolling patients before implementation and following them for 12 months for outcome assessment (e-Cohort).

#### Sample size

Based on preliminary data from the diagnostic phase and published literature, the baseline rate of achievement for the composite primary outcome (glycaemic control (HbA1c <8%); blood pressure control (<140/90 mm Hg); and use of statins (statin prescription) -limited to patients ≥40 years-) in the control group is estimated at 30%. The intervention is expected to improve this to ≥50% (absolute effect size of 20%). We have calculated the sample size considering a parallel cluster randomised trial with a cohort design, including baseline and follow-up assessments. The trial is powered at 80% to detect an absolute difference of 20 percentage points between the control group (30%) and the intervention group (50%) on the composite primary outcome, assuming a two-sided significance level of 5%. The design includes six clusters per arm (12 in total) and accounts for the correlation structure of repeated measures using a two-period decay model. Specifically, we assumed an intracluster correlation coefficient (ICC) of 0.05, a cluster autocorrelation of 0.5, and an individual autocorrelation of 0.6. Standard errors were adjusted using a t-distribution to account for the small number of clusters. Based on these parameters, the required sample size before adjusting for attrition is 312 individuals (26 per cluster). Applying an attrition inflation factor of 1.25 (to allow for 20% loss to follow-up), the final sample size increases to 396 individuals (33 per cluster, 198 per arm).

#### Randomisation procedures

Clusters will be randomly assigned in a 1:1 ratio to the intervention or control arm following completion of baseline data collection. A restricted randomisation approach will be used to ensure balance across arms with respect to baseline HbA1c levels, key sociodemographic characteristics (such as age, sex, income and education), and cluster size. These parameters will be used to guide the allocation process to maintain equivalence across study arms, thereby strengthening the internal validity of the research. Should any residual imbalances arise post-randomisation, we will adjust for these covariates in the statistical analyses.

#### Implementation

The PHC care model and its associated implementation strategies, codesigned and refined during Phase I, will be deployed in the six clusters allocated to the intervention arm. Control clusters will continue to deliver usual care and will be offered the intervention package after the trial if it proves effective. Intervention clusters will participate in a Quality Improvement Learning Network (QILN), a collaborative platform designed to foster shared learning, experimentation and continuous improvement in care delivery and outcomes.^30^ The QILN will be delivered through virtual activities, including quarterly online sessions involving all intervention sites (‘full-house’ meetings), monthly 30 min group outreach sessions focused on emerging implementation challenges, and monthly 1-hour multisite meetings to support peer learning and problem-solving. Sessions will be facilitated by trained study coordinators and local experts and will combine brief structured inputs with facilitated discussion to support experimentation, reflection and exchange of experiences across centres. The QILN is designed to be embedded within existing professional routines and relies on low-cost virtual platforms, facilitating continuation of peer-learning activities beyond the trial period if the intervention proves effective.

#### Outcomes

The primary outcome will be a composite outcome including glycaemic control (HbA1c <8%); blood pressure control (<140/90 mm Hg); and use of statins (statin prescription) limited to patients ≥40 years. The source to obtain this data will be routine clinical records.

Secondary outcomes measured through participant questionnaires include user-reported quality of care (key secondary outcome), PHC engagement, system competence, patient confidence in the health system, inequities in outcomes and patient empowerment. User-reported Quality of Care is the overall rating of quality of care from enrolled patients, categorised as ‘excellent or very good’ versus ‘good’ or worse. PHC engagement is measured as the percentage of visits conducted at the PHC level during the data collection period, with positive engagement defined as ≥75% of all non-urgent ambulatory visits.^31^ System competence is defined as ≥75% of enrolled patients per site adhering to the complete set of selected diabetes prevention recommendations from national and provincial guidelines: annual eye exam, yearly foot exam, annual kidney function assessment, annual blood pressure measurement and biannual HbA1c measurement during the follow-up period. Patient confidence in the health system is measured by the combination of three aspects in the e-Cohort survey: health security, endorsement of the current system and trajectory.^32^ Security: We will calculate the percentage of respondents answering ‘somewhat’ or ‘very confident’ to ‘How confident are you that you would receive good quality healthcare if you became very sick?’ and ‘that you could afford the healthcare you needed if you became very sick?’. Confidence: The proportion of respondents indicates that in the past 2 years, the health system has been improving (vs staying the same or getting worse). Endorsement is indicated by agreement that the system works well and needs only minor reforms (vs significant changes needed or need to be completely rebuilt). To assess inequities, we will explore gender, migration status, education and income as potential variables related to underperformance in primary outcomes. Finally, we will measure patient empowerment using the Diabetes Empowerment Scale. This instrument is free and easy to use and has been adapted and validated in its Spanish version.^33^

Direct costs related to diabetes care: Estimated from costs assessed included medications, diagnostic tests, procedures, medical supplies and visits with health professionals. Results will be reported on average per outpatient visit in USD using Purchasing Power Parity Index from ARS. Source: Accounting Department of Ministry of Health.

#### Implementation process outcomes

We will conduct an integrated process evaluation using a mixed-methods approach, including semistructured interviews, focus group discussions, surveys, documentary analysis and direct observations. Guided by NPT, this evaluation will explore how implementers engage with the selected implementation strategies and how contextual factors influence their efforts to drive improvement. Specifically, we will examine the extent to which the strategies are delivered as intended (fidelity of delivery), how they are understood and accepted by recipients (fidelity of receipt), and how they are applied in practice (fidelity of enactment). This approach will allow us to assess the mechanisms through which the intervention becomes embedded in routine practice and identify factors that facilitate or hinder its normalisation.

#### Data collection

We will construct a longitudinal cohort of patients with T2D in both arms, enrolling patients before implementation and following them for 12 months for outcome assessment and an additional 6 months for sustainability assessment. Our methodology builds on the e-Cohort framework that we previously optimised and used for this context at the provincial level.^34^ Participants will be contacted via telephone or in person at the PHC centre for eligibility assessment. If eligible, they will have their first in-person visit at their PHC centre, during which informed consent will be requested for recruitment (onlinesupplemental materials A  B). For all consenting patients, we will extract data about patient visits and laboratory testing from routine clinical records and costs from administrative databases for all participating facilities. During the first visit, we will administer a baseline questionnaire. Subsequently, we will conduct quarterly telephone surveys and conclude with an endline questionnaire in person at the end of the intervention period. We will implement a standardised clinical electronic data collection system using REDCap for all study data collection phases. The research team will collect monitoring data from participating facilities throughout the trial. This includes attendance at QILN activities (total attendance and sustained participation by initial team members), team meetings within each facility, timely submission of implementation team reports and implementation checklists completed quarterly during the study team observations. Specially trained data collectors will perform daily data processing at each participating PHC centre.

### Phase III: analysis and reporting

#### Objectives

The objectives of this phase are (1) to evaluate the effectiveness of the intervention and its sustainability, (2) to report findings and (3) to engage stakeholders in disseminating results and translating their results to public policy.

#### Procedures

Procedures in this phase will include (a) Analysis of findings: Once the data management team closes the database, we will conduct the analysis plan as described below, (b) Report on study results: The main manuscripts on the different study phases will be written and sent out for peer review before the closure of the grant period. Engagement of policymakers through workshops will be conducted to interpret results in context. See more in dissemination below, (c) Control arm clusters will receive the intervention, if proven effective, after the study follow-up period, but prior to the completion of data analysis and (d) Communicate study results to stakeholders to promote policy adoption.

#### Data analysis

We will use all available data from all randomised clusters and conduct all analyses on the intention-to-treat (ITT) population, including all clusters and patients in the randomised group, regardless of intervention adherence. Summary statistics will be given for baseline data by treatment group using means, SD, medians, minimum, maximum and quartiles for continuous variables and counts and percentages for categorical variables. We will quantify complete vs incomplete records and describe the characteristics of patients lost to follow-up.

For the composite clinical outcome, the primary ITT analysis will compare the proportion of patients achieving an HbA1c level <8%, blood pressure control <140/90 mm Hg and statin uptake between the trial arms at the end of the study. We expect a good covariate balance between the groups; therefore, the ITT analysis will be performed by comparing proportions. Patient and cluster-level covariates can be included in a mixed-effects logistic regression model to account for the cluster effect and obtain an adjusted estimate of the average treatment effect for within-cluster change.^35^ Adjusted ORs will be reported with 95% CIs, p values and ICCs will be reported to estimate the percentage of variance explained by each level.

For quality of care, we will compare the proportion of patients reporting overall quality of care as ‘excellent’ or ‘very good’ before and after the intervention phase between trial arms using difference-in-difference regression analysis to estimate the average treatment effect. Engagement and confidence in the health system will be evaluated by comparing the proportion of patients achieving the expected threshold in each trial using a χ^2^ test for proportions. Fisher’s exact test will evaluate system competence across participating PHC centres. Direct costs related to diabetes care will be reported for each group. We may expect higher costs in the intervention group due to increased and better utilisation of health services. The mean cost will be analysed considering the variable distribution (non-normal/normal). For all outcomes, we will conduct mixed effects regression adjusted for covariates and clustered by department to provide an average treatment effect for expected change in outcomes within each department.

We will use multiple approaches to address missing data. For clinical outcomes, those with missing outcome data will be considered as not achieving the composite outcome for primary analyses, given the lack of evidence of disease control. We will conduct a sensitivity analysis using inverse probability weights calculated from clinical and participant data to reweight the complete data sample to the total patient population. Inverse weights provide valid estimates of the full sample estimate if the assumption of missing at random is met. We will apply the same approach for secondary outcomes. Based on experience with the eCohort methodology, we anticipate high response rates and largely complete responses within the survey. In the event of greater than anticipated missingness within surveys (eg, skipped items), we will conduct an additional analysis using multiple imputation approaches to robustly impute missing responses.^36^

Planned subgroup analyses will explore the potential differential effects of the intervention by gender, migrant status, income and educational level (effect modifiers). This will indicate whether the intervention contributes to reducing inequalities in care. Subgroup analyses will be exploratory. To evaluate inequalities, we will estimate the slope index of inequality and the absolute gradient of inequality across income groups and educational attainment to quantify the absolute inequality in each outcome within the sample in each arm. All analyses will be performed using R V.4.3.1^37^

## Ethics and dissemination

All study activities will comply with national and international ethics guidelines, presenting minimal risk to participants. The protocol was submitted and approved by the local independent ethics committee at the Mendoza Ministry of Health (CoPEIS—Consejo Provincial de Evaluación ética en investigación). Facility-level permission will be obtained for participation and sharing of deidentified data. Written informed consent will be required from study participants (patients with T2D who meet the eligibility criteria), who will receive information on the study’s purpose, procedures, risks and benefits. Evaluation forms, surveys and records will use coded identifiers to ensure confidentiality. Records will be securely stored, and clinical information will only be released with written participant permission or for regulatory monitoring.

Dissemination activities and outputs will include writing and submitting manuscripts for publication; writing policy briefs to support strategy implementation in other regions or countries; and tailoring outputs for patients, clinicians and researchers. We anticipate that improvements in disease management and patient experience will have clinical and economic benefits related to reduced usage of secondary-level and tertiary-level facilities, lower cost per visit and a reduced number of clinical events related to diabetes. On a larger scale, this project constitutes one of the first experiences in SDR for NCDs, and its findings will be informative for future work in this line of research and health policy worldwide with a particular focus on LMICs.

## Supplementary material

10.1136/bmjopen-2025-111459 online supplemental file 1

10.1136/bmjopen-2025-111459 online supplemental file 2
