# Supplementary material for: Cluster randomised controlled trial for service delivery redesign of primary care for people with diabetes: study protocol
Source: BMJ Open. 2026 Mar 18;16(3):e111459. doi: 10.1136/bmjopen-2025-111459 (PMC13007129; doi:10.1136/bmjopen-2025-111459)
Supplement: online supplemental file 1 [file bmjopen-16-3-s001.pdf]

CONSENTIMIENTO INFORMADO

HOJA INFORMATIVA

- Pacientes –

Investigador principal: Dr. Ezequiel García Elorrio

Título del estudio: Servicio de Atención Rediseñado para personas con Diabetes: ensayo clínico aleatorizado. SARA-D

Institución responsable: Instituto de Efectividad Clínica y Sanitaria – IECS – Buenos Aires

Financiado por: NIHR número 158215

¿Sobre qué es la investigación?

Se lo invita a participar voluntariamente en un estudio de investigación para evaluar la implementación de un modelo de atención rediseñado para pacientes con diabetes. El propósito del estudio es mejorar el manejo de la diabetes y la experiencia de los usuarios con la enfermedad en el primer nivel de atención. La información proporcionada en este documento lo ayudará a decidir si usted desea participar en nuestro estudio de investigación. Si hay algo que usted no entiende, o si necesita obtener más información, por favor pregúntenos, y con gusto se lo aclararemos. Tómese el tiempo que necesite para decidir si desea participar. Si usted acepta, se le pedirá que firme este formulario de consentimiento y se le dará una copia para que guarde.

El estudio está siendo conducido por investigadores del Instituto de Efectividad Clínica y Sanitaria (IECS) (Argentina), el Ministerio de Salud de la provincia de Mendoza y la Red de Transformación de Sistemas de Evidencia de Calidad de Latinoamérica y el Caribe (Red QuEST-LAC). La red QuEST LAC se trata de un grupo de investigación sobre la calidad de los sistemas de salud lanzado por la Escuela de Salud Pública de Harvard en Estados Unidos. En Latinoamérica y el Caribe, esta red de investigadores está liderada por el IECS en Buenos Aires, Argentina y la Universidad Peruana Cayetano Heredia, en Lima, Perú.

Potencial conflicto de interés

El IECS es una institución académica sin fines de lucro. Los/as investigadores/as de este estudio son profesionales de la salud y están interesados/as en su bienestar y en los conocimientos que se

puedan obtener de este estudio. Los/as investigadores/as reciben un salario para conducir el estudio.

¿Por qué se realiza este estudio?

La razón principal por la que estamos haciendo este estudio es implementar un modelo de atención rediseñado para mejorar la calidad de la atención y los resultados en salud de las personas con diabetes.

¿Quién está siendo invitado a participar en este estudio?

Pacientes adultos con diagnóstico de diabetes tipo 2

¿Tengo obligación de participar en el estudio?

No. Usted puede decidir si desea participar o no en este estudio. Vamos a explicarle los detalles del estudio con el fin de responder a cualquier pregunta que pueda tener. Si acepta participar y luego cambia de opinión, podrá retirarse del estudio en cualquier momento que desee sin tener que explicar los motivos de su decisión. Su atención médica no va a ser perjudicada por participar en el estudio.

¿Qué pasa si decido participar en este estudio?

Si decide participar, investigadores/as del IECS tomarán datos sobre visitas médicas y pruebas de laboratorio de su historia clínica (incluida hemoglobina glicosilada) sin que se pueda identificar a quienes pertenecen.

A su vez, lo invitaremos a concurrir a su centro de salud habitual y se le realizará un cuestionario con preguntas relacionadas a su enfermedad. Durante el estudio podrá recibir la atención habitual o bien recibir invitaciones a participar en algún taller informativo, en grupos de apoyo, recibir algún recordatorio de su próxima visita.

Además, se lo contactará telefónicamente cada dos meses para realizar un simple seguimiento sobre su tratamiento de la diabetes. Al cabo de unos meses, también le haremos unas preguntas durante su visita al centro médico sobre su salud y la atención recibida. Estos cuestionarios no son una evaluación de sus conocimientos. Los datos obtenidos permitirán analizar cómo las personas usan el sistema de salud y cuáles son sus experiencias con el manejo de su enfermedad con el objetivo de generar una mejor atención en el futuro. Al finalizar el estudio, todos los participantes,

incluso aquellos que en un principio estuvieron en el grupo que no recibían atención según el nuevo modelo, recibirán el nuevo modelo de atención de modo completo.

¿Cuánto dura el estudio y donde se realizará?

El estudio durará 30 meses y se incluirán ocho departamentos de la provincia de Mendoza, Argentina.

¿Cuáles son los posibles riesgos y molestias por participar en este estudio?

El estudio no tiene riesgos. El personal del centro de salud puede hacerle preguntas que usted considere incómodas de responder, pero en ese caso usted no está obligado a responder y siempre será guardando la privacidad de sus respuestas.

¿Hay beneficios por participar en este estudio?

Esperamos que esta investigación nos ayude a mejorar la calidad de la atención y los resultados en salud de las personas con diabetes. No habrá beneficios económicos directos para los pacientes que participen en este estudio.

¿Qué pasará con los resultados obtenidos en el estudio?

Los resultados del estudio serán publicados en revistas médicas y serán informados a las autoridades del Ministerio de Salud a fin de trasladarlos a mejoras en políticas públicas. No se incluirá ningún dato que permita identificar a las personas que participaron del estudio ni al equipo de salud que los atiende.

¿Tendrá algún costo participar de este estudio?

No hay costos para usted. Su participación en este estudio no le generará ningún gasto.

¿Quién podrá ver la información personal recopilada en el estudio?

Sólo los/las investigadores/as de este estudio van a tener acceso a la información recogida durante el estudio. Toda la información que usted proporcione es estrictamente confidencial. La información en papel y los archivos electrónicos estarán protegidos con una clave de acceso. El formulario de consentimiento informado se mantendrá en un lugar seguro, y sólo el equipo de investigación podrá verlo. Toda otra información (su edad, sexo, fecha de nacimiento) se hará

anónima utilizando un código para que, cuando los resultados se analicen, estos datos no se asocien con su nombre.

Todos los datos serán guardados siguiendo normas éticas sobre investigación. El investigador principal se hace cargo del resguardo de la información, que no haya filtración de nombres o características que permitan identificar al entrevistado/a, y es responsable de destruir la información una vez cumplido el lapso de resguardo. Esta información se guardará por tres meses a partir de la finalización del estudio.

Toda la información personal será destruida al final del estudio.

De acuerdo con la Ley 25.326, usted tiene derecho a acceder a sus datos personales sin ningún costo. Además, tiene derecho a solicitar la corrección de sus datos. La Dirección Nacional de Protección de Datos Personales es el órgano de control de la Ley 25.326 (Dirección: Sarmiento 1118, 5º piso, C1041AAX CABA, tel. 011-4383-8512/13, email: infodnpd@jus.gov.ar).

¿Quién está a cargo de este estudio?

El estudio está coordinado por el Instituto de Efectividad Clínica y Sanitaria (IECS) y Dr. Ezequiel García Elorrio, quien es el investigador principal. En la provincia de Mendoza, las co investigadoras principales, Dra. Andrea Falaschi y Dra. Yanina Mazzaresi.

Evaluación del consentimiento informado

El Consejo Provincial de Evaluación Ética de Investigaciones en Salud (COPEIS), de la Provincia de Mendoza, evaluó el protocolo de investigación y el consentimiento informado en cuanto al cumplimiento de normas éticas, como así también la coherencia entre el proyecto de investigación y el consentimiento informado.

Financiación

El estudio será financiado por The National Institute for Health and Care Research (NIHR por sus siglas en inglés) número 158215.

¿Qué pasa si tengo más preguntas?

Si usted desea comunicarse con un miembro de nuestro equipo de investigación en el futuro, puede comunicarse con la Dra. Yanina Mazzaresi al teléfono +549 261662 8638 o al correo electrónico yaninamazzaresi@gmail.com, Ministerio de Salud, Desarrollo Social y Deportes de la

provincia de Mendoza, o llamar al teléfono del Instituto de Efectividad Clínica y Sanitaria: 011 4777-8767, Dr. Ezequiel García-Elorrio, o al correo electrónico egarciaelorrio@iecs.org.ar. Si tiene preguntas sobre los aspectos éticos del estudio, o cree que ha sido tratado injustamente, puede contactar al Consejo Provincial de Evaluación Ética de Investigaciones en Salud (COPEIS), de la Provincia de Mendoza, al número de teléfono (0261)-4234425 y/ o comunicarse al siguiente correo electrónico fpalmans@mendoza.gov.ar. Se le pide que firme un formulario de consentimiento informado (que está incluido aquí) en el que se explica que usted está de acuerdo con participar.

FORMULARIO DE CONSENTIMIENTO INFORMADO PARA PACIENTES

Fecha: ______________

YO,____

(Nombre del participante)

He leído este formulario y he decidido participar en el estudio mencionado anteriormente. Fui informado sobre el propósito del estudio, los detalles de la participación, los beneficios, los posibles riesgos e inconvenientes que puedan presentarse. Comprendo que puedo retirarme del estudio en cualquier momento. Mi firma también indica que he recibido un original del presente formulario de consentimiento.

_____________________________ __________________ ________________

Firma del participante Aclaración Fecha

He explicado los objetivos y métodos de este estudio al participante cuyo nombre aparece arriba.

_____________________________ __________________ ________________

Firma del investigador/a Aclaración Fecha

_____________________________ __________________ ________________

Firma del testigo Aclaración Fecha
