# Supplementary material for: Cluster randomised controlled trial for service delivery redesign of primary care for people with diabetes: study protocol
Source: BMJ Open. 2026 Mar 18;16(3):e111459. doi: 10.1136/bmjopen-2025-111459 (PMC13007129; doi:10.1136/bmjopen-2025-111459)
Supplement: online supplemental file 2 [file bmjopen-16-3-s002.pdf]

# Informed Consent Form

**INFORMATION SHEET**

**- Patients –**

**Principal investigator:** Dr Ezequiel García Elorrio

**Study title:** Redesigned Care Service for People with Diabetes: Randomised Clinical Trial. SARA-D

**Responsible institution:** Institute for Clinical and Health Effectiveness – IECS – Buenos Aires

**Funded by:** NIHR number 158215

**What is the research about?**

You are invited to voluntarily participate in a research study to evaluate the implementation of a redesigned care model for patients with diabetes. The purpose of the study is to improve diabetes management and the experience of users with the disease in primary care. The information provided in this document will help you decide whether you wish to participate in our research study. If there is anything you do not understand, or if you need more information, please ask us, and we will be happy to clarify it for you. Take as much time as you need to decide whether you wish to participate. If you agree, you will be asked to sign this consent form and will be given a copy to keep.

The study is being conducted by researchers from the Institute for Clinical and Health Effectiveness (IECS) (Argentina), the Ministry of Health of the province of Mendoza, and the Latin American and Caribbean Quality Evidence Systems Transformation Network (Red QuEST-LAC). The QuEST LAC network is a research group on the quality of health systems launched by the Harvard School of Public Health in the United States. In Latin America and the Caribbean, this network of researchers is led by the IECS in Buenos Aires, Argentina, and the Cayetano Heredia Peruvian University in Lima, Peru.

**Potential conflict of interest**

The IECS is a non-profit academic institution. The researchers in this study are health professionals who are interested in their well-being and in the knowledge that can be gained from this study. The researchers receive a salary for conducting the study.

**Why is this study being conducted?**

The main reason we are conducting this study is to implement a redesigned care model to improve the quality of care and health outcomes for people with diabetes.

**Who is being invited to participate in this study?**

Adult patients diagnosed with type 2 diabetes

**Am I obliged to participate in the study?**

No. You can decide whether or not you want to participate in this study. We will explain the details of the study to you in order to answer any questions you may have. If you agree to participate and then change your mind, you can withdraw from the study at any time without having to explain your reasons for doing so. Your medical care will not be affected by your participation in the study.

**What happens if I decide to participate in this study?**

If you decide to participate, IECS researchers will collect data on medical visits and laboratory tests from your medical records (including glycosylated haemoglobin) without identifying who they belong to.

We will also invite you to visit your usual health centre, where you will be asked to complete a questionnaire about your condition. During the study, you will receive your usual care or invitations to participate in informational workshops or support groups, as well as reminders about your next visit.

In addition, you will be contacted by telephone every two months for a simple follow-up on your diabetes treatment. After a few months, we will also ask you some questions during your visit to the medical centre about your health and the care you have received. These questionnaires are not an assessment of your knowledge. The data obtained will allow us to analyse how people use the health system and what their experiences are with managing their disease, with the aim of providing better care in the future. At the end of the study, all participants, including those who were initially in the group that did not receive care under the new model, will receive the new model of care in its entirety.

**How long will the study last and where will it be conducted?**

The study will last 30 months and will include eight departments in the province of Mendoza, Argentina.

**What are the possible risks and discomforts of participating in this study?**

The study has no risks. The health centre staff may ask you questions that you find uncomfortable to answer, but in that case, you are not obliged to answer and the privacy of your answers will always be protected.

**Are there any benefits to participating in this study?**

We hope that this research will help us improve the quality of care and health outcomes for people with diabetes. There will be no direct financial benefits for patients who participate in this study.

**What will happen to the results obtained in the study?**

The results of the study will be published in medical journals and reported to the Ministry of Health authorities in order to translate them into improvements in public policy. No data will be included that could identify the people who participated in the study or the healthcare team that treats them.

**Will there be any cost to participate in this study?**

There are no costs to you. Your participation in this study will not incur any expenses.

**Who will be able to see the personal information collected in the study?**

Only the researchers involved in this study will have access to the information collected during the study. All information you provide is strictly confidential. Paper and electronic files will be protected with a password. The informed consent form will be kept in a secure location and only the research team will be able to view it. All other information (your age, gender, date of birth) will be anonymised using a code so that when the results are analysed, this data will not be associated with your name.

All data will be stored in accordance with ethical research standards. The principal investigator is responsible for safeguarding the information, ensuring that no names or characteristics that could identify the interviewee are disclosed, and for destroying the information once the retention period has expired. This information will be stored for three months after the study is completed.

All personal information will be destroyed at the end of the study.

In accordance with Law 25.326, you have the right to access your personal data at no cost. You also have the right to request the correction of your data. The National Directorate for Personal Data

Protection is the supervisory body for Law 25,326 (Address: Sarmiento 1118, 5th floor, C1041AAX CABA, tel. 011-4383-8512/13, email:infodnpd@jus.gov.ar ).

**Who is in charge of this study?**

The study is coordinated by the Institute for Clinical and Health Effectiveness (IECS) and Dr. Ezequiel García Elorrio, who is the principal investigator. In the province of Mendoza, the co-investigators are Dr. Andrea Falaschi and Dr. Yanina Mazzaresi.

**Informed consent assessment**

The Provincial Council for the Ethical Evaluation of Health Research (COPEIS) of the Province of Mendoza evaluated the research protocol and informed consent in terms of compliance with ethical standards, as well as the consistency between the research project and the informed consent.

**Funding**

The study will be funded by The National Institute for Health and Care Research (NIHR) number 158215.

**What if I have more questions?**

If you would like to contact a member of our research team in the future, you can contact Dr Yanina Mazzaresi on +549 261662 8638 or by email at yaninamazzaresi@gmail.com , Ministry of Health, Social Development and Sports of the Province of Mendoza, or call the Institute for Clinical and Health Effectiveness on 011 4777-8767, Dr Ezequiel García-Elorrio, or email egarciaelorrio@iecs.org.ar . If you have questions about the ethical aspects of the study, or believe you have been treated unfairly, you may contact the Provincial Council for Ethical Evaluation of Health Research (COPEIS) of the Province of Mendoza at (0261)-4234425 and/or email fpalmans@mendoza.gov.ar . You will be asked to sign an informed consent form (included here) explaining that you agree to participate.

## **INFORMED CONSENT FORM FOR PATIENTS**

Date: _____________

I,__________________________________________________________________(name)

I have read this form and have decided to participate in the above-mentioned study. I have been informed about the purpose of the study, the details of participation, the benefits, and the possible risks and drawbacks that may arise. I understand that I may withdraw from the study at any time. My signature also indicates that I have received an original copy of this consent form.

___________________________ ________________ _______________

Participant's signature Clarification Date

I have explained the objectives and methods of this study to the participant whose name appears above.

___________________________ ________________ _______________

Signature of the researcher Clarification Date

___________________________ ________________ _______________

Witness signature Clarification Date
