# Supplementary material for: Cluster randomised controlled trial for service delivery redesign of primary care for people with diabetes: study protocol
Source: BMJ Open. 2026 Mar 18;16(3):e111459. doi: 10.1136/bmjopen-2025-111459 (PMC13007129; doi:10.1136/bmjopen-2025-111459)
Supplement: Supplementary file 3 [file bmjopen-2025-111459.reviewer_comments.pdf]

PEER REVIEW HISTORY

BMJ Open publishes all reviews undertaken for accepted manuscripts. Reviewers are asked to complete a checklist review form and are provided with free text boxes to elaborate on their assessment. These free text comments are reproduced below.

ARTICLE DETAILS

Title (Provisional)

Cluster randomised controlled trial for service delivery redesign of primary care for people with diabetes: Study protocol

Authors

Mazzoni, Agustina; Roberti, Javier; Guglielmino, Marina; Jorro-Baron, Facundo; Mazzaresi, Yanina; Falaschi, Andrea; Alvarez, Jorgelina; Gibbons, Luz; Leslie, Hannah; Silva, Cecilia; Garcia, Patricia J; Garcia Elorrio, Ezequiel

---

VERSION 1 - REVIEW

**Reviewer**	**1**
**Name**	**Ching, Siew Mooi**
**Affiliation**	**Universiti Putra Malaysia, Family Medicine**
**Date**	**26-Dec-2025**
**COI**	**None**

Thank you for inviting me to review the protocol: Abstract

1) 6-month sustainability period or 6 months follow-up period

Implementation Strategies Detail (page 13, line 25..)

Q1 I am NOT clear on the “Quality Improvement Learning Network”. Please elaborate in detail on this network e.g. what specific online activities will be designed to foster shared learning, experimentation, and continuous improvement in care delivery and outcomes. How the assessment be measured and sustained even though the study has been completed? Any extra cost incurred for the maintenance in terms of the manpower ?

Q2 Sustainability Assessment: You plan a 6-month sustainability follow-up. What specific measures are you going to take and whether the redesigned care model and its strategies are sustained after the trial has been completed?

Q3 Missing Data session,pg 18 line 6: “Although we expect the level of missing data to be minor, we will investigate patterns and reasons for missing data”. Any possibility to handle the missing data using other method e.g., multiple imputation if the percentage of missing data is less than 5%?

Q4 Generalizability and Adaptation, page 5 line 6: "Implementing this study in an LMIC is complex and influenced by dynamic factors such as political shifts, workforce constraints, competing policy priorities, and resource limitations. Findings from a single province in Argentina may have limited generalizability to other settings without careful adaptation to local health system structures and sociocultural dynamics"

Q5 My question is what are the measures are you going to take so that the implementation of the new model will support the adaptation in real clinical or other LMIC settings if they have no budget to conduct a similar study.

Q6 Analysis: Any role by using GEE Analysis instead of mixed logistic analysis?

VERSION 1 - AUTHOR RESPONSE

Reviewer: 1

Abstract

1) 6-month sustainability period or 6 months follow-up period

***Response**: We have revised the abstract and main text to use the term "sustainability period" consistently throughout the document. We have also replaced the term "follow-up period" with "intervention period" to avoid confusion.*

Implementation Strategies Detail (page 13, line 25..)
Q1 I am NOT clear on the "Quality Improvement Learning Network". Please elaborate in detail on this network e.g. what specific online activities will be designed to foster shared learning, experimentation, and continuous improvement in care delivery and outcomes. How the assessment be measured and sustained even though the study has been completed? Any extra cost incurred for the maintenance in terms of the manpower?

***Response**: Thank you for this comment. We appreciate the opportunity to clarify the structure, activities, assessment, and sustainability of the Quality Improvement Learning Network (QILN).*

The QILN is a collaborative implementation support platform designed to enable participating sites to collectively learn, test, and share experiences related to the implementation strategies, with the aim of improving care delivery and patient outcomes. It is not a standalone intervention, but a mechanism to support continuous quality improvement during the implementation phase.

The QILN will be delivered through a series of virtual components, including:

- *Quarterly online "full-house" sessions bringing together all intervention sites to share experiences, review progress, and discuss common challenges*
- *Monthly 30-minute virtual group outreach sessions focused on emerging implementation issues and peer-to-peer learning; and*
- *Monthly one-hour virtual multisite meetings to support deeper discussion, reflection, and problem-solving across centres.*

These activities combine brief structured inputs with facilitated discussion, enabling participants to reflect on implementation experiences, test adaptations in their local context, and share lessons learned across sites. Trained study coordinators and local experts will oversee and facilitate these sessions, supporting experimentation, shared learning, and continuous improvement.

Regarding the assessment and sustainability of the QILN, the contribution of the QILN will be assessed as part of the overall process evaluation rather than as a separate effectiveness outcome. Assessment will draw on multiple data sources, including attendance records, session documentation, monitoring reports, and qualitative data from interviews and focus groups with healthcare professionals and coordinators.

The QILN is designed to be embedded within existing professional and institutional routines, using low-cost virtual platforms and peer-learning mechanisms. If the intervention proves effective, participating sites will be able to sustain similar learning and exchange activities beyond the trial period without reliance on project-specific infrastructure. The QILN does not incur substantial additional costs. It is delivered through existing virtual communication tools and facilitated by study coordinators and local experts already funded within the project.

In response to this comment, we have updated the manuscript by expanding the description of the QILN in the Methods and analysis section, including details on its virtual activities, integration within the process evaluation, and sustainability beyond the trial period.

Q2 Sustainability Assessment: You plan a 6-month sustainability follow-up. What specific measures are you going to take and whether the redesigned care model and its strategies are sustained after the trial has been completed?

Response*: The 6-month sustainability period is intended to assess whether the redesigned care model and its core implementation strategies are maintained after the intervention phase. Sustainability will be examined as part of the process evaluation, focusing on continuation and integration of key practices rather than long-term clinical outcomes.*

Specifically, we will assess the ongoing use of selected implementation strategies (e.g. protected appointments, diabetes patient registry, WhatsApp communication, and group workshops), their integration into routine workflows, and levels of professional and organizational engagement. Data sources will include routine monitoring records and documentation collected during the sustainability period, analysed using the same theoretical framework applied in the process evaluation.

Sustainability beyond the trial will be supported through capacity building and embedding low-cost strategies within existing systems. No additional data collection tools or resources are required for the sustainability assessment.

Q3 Missing Data session, pg 18 line 6: "Although we expect the level of missing data to be minor, we will investigate patterns and reasons for missing data". Any possibility to handle the missing data using other method e.g., multiple imputation if the percentage of missing data is less than 5%?

Response: *We appreciate this suggestion and have clarified and consolidated description of the handling of missing data within the revised manuscript. The revised paragraph (p16) reads: We will use multiple approaches to address missing data. For clinical outcomes, those with missing outcome data will be considered as not achieving the composite outcome for primary analyses, given the lack of evidence of disease control. We will conduct a sensitivity analysis using inverse probability weights calculated from clinical and participant data to reweight the complete data sample to the total patient population. Inverse weights provide valid estimates of the full sample estimate if the assumption of missing at random is met. We will apply the same approach for secondary outcomes. Based on experience with the eCohort methodology, we anticipate high response rates and largely complete responses within survey. In the event of greater than anticipated missingness within surveys (e.g., skipped items), we will conduct an additional analysis using multiple imputation approaches to robustly impute missing responses.*

Q4 Generalizability and Adaptation, page 5 line 6: "Implementing this study in an LMIC is complex and influenced by dynamic factors such as political shifts, workforce constraints, competing policy priorities, and resource limitations. Findings from a single province in Argentina may have limited generalizability to other settings without careful adaptation to local health system structures and sociocultural dynamics"

Response*: As discussed in the manuscript, we recognise that implementing complex service delivery interventions in LMICs is strongly shaped by contextual factors, including political dynamics, workforce constraints, and resource availability. Accordingly, our aim is not to claim direct transferability of findings from a single province in Argentina, but to support analytical generalisability.*

The study uses a theory-informed approach (Normalisation Process Theory) and provides detailed reporting of context, implementation processes, adaptations, and mechanisms of action. This allows other LMIC settings to assess relevance, identify comparable challenges, and adapt the intervention to local health system structures and sociocultural conditions.

Q5 My question is what are the measures are you going to take so that the

implementation of the new model will support the adaptation in real clinical or other LMIC settings if they have no budget to conduct a similar study.

Response: *Thank you for this question. The intervention package was intentionally designed to minimise additional costs and to rely primarily on reorganisation of existing resources rather than new infrastructure or funding. Most implementation strategies (e.g., protected appointments, community resource mapping, group education, WhatsApp communication) can be implemented using existing staff, routine workflows, and widely available technologies.*

Adaptation in settings without resources to conduct a similar study is supported through clear specification of core versus adaptable components, detailed documentation of implementation processes and barriers, and use of implementation theory to guide local decision-making. Training materials, monitoring tools, and implementation guidance developed during the study are designed to be reusable, supporting pragmatic uptake in routine clinical practice without the need for additional research funding.

Q6 Analysis: Any role by using GEE Analysis instead of mixed logistic analysis?

Response*: Thank you for this suggestion. We appreciate the role of GEE models in providing between cluster estimates of the ATE; in this case, given the relatively large clusters (departments), we prioritize mixed effects models for quantifying the expected difference between intervention state and control state within-cluster in addition to providing a direct estimate of the variance components by level. Mixed models provide the additional benefit of allowing for clustering within department and within facility within department, should we identify clustering at each level. We have noted the conceptual basis for selection of mixed effect models within the manuscript, stating for example: "For all outcomes, we will conduct mixed effects regression adjusted for covariates and clustered by department to provide an average treatment effect for expected change in outcomes within each department."*
